# Oral communication in individuals with hearing impairment—considerations regarding attentional, cognitive and social resources

**DOI:** 10.3389/fpsyg.2015.00998

**Published:** 2015-07-17

**Authors:** Ulrike Lemke, Sigrid Scherpiet

**Affiliations:** Cognitive and Ecological Audiology, Science and Technology, Phonak AG, Stäfa, Switzerland

**Keywords:** communication, hearing impairment, executive functions, cognitive aging, speech comprehension, third-party disability

## Abstract

Traditionally, audiology research has focused primarily on hearing and related disorders. In recent years, however, growing interest and insight has developed into the interaction of hearing and cognition. This applies to a person’s listening and speech comprehension ability and the neural realization thereof. The present perspective extends this view to oral communication, when two or more people interact in social context. Specifically, the impact of hearing impairment and cognitive changes with age is discussed. In focus are executive functions, a group of top-down processes that guide attention, thought and action according to goals and intentions. The strategic allocation of the limited cognitive processing capacity among concurrent tasks is often effortful, especially under adverse communication conditions and in old age. Working memory, a sub-function extensively discussed in cognitive hearing science, is here put into the context of other executive and cognitive functions required for oral communication and speech comprehension. Finally, taking an ecological view on hearing impairment, activity limitations and participation restrictions are discussed regarding their psycho-social impact and third-party disability.

## General Aspects of Oral Communication

Being able to communicate with others is regarded a key element of human functioning. During oral communication individuals interact with each other, and also with their social and physical surroundings by exchanging information in form of language, signals, and behavior (Stephens and Kramer, 2009). As such oral communication constitutes by far a more complex process than serving the basic purpose of sending and receiving information. Communication implies bidirectional transfer of information, meaning, and intent between two or more individuals (Kiessling et al., 2003). As such, it is a social act originating from the need to express oneself, and to relate to others. Furthermore, interactions are mediated by psychological variables of the communication partners such as emotions, attitudes, and beliefs as well as by values and rules of the community. Thus, oral communication is a broad concept encompassing perceptual, cognitive, psychological, and social constructs.

Hearing impairment constitutes a major challenge in this respect as it generally leads to difficulties in oral communication (Stephens and Jones, 2005). These communication problems are often age-related and accompanied by impairment of other sensory modalities and comorbid health problems (Kramer et al., 2002; Davis and Davis, 2009; Lemke, 2009; Stam et al., 2014). Age-related hearing impairment (presbycusis) begins in the fourth decade and its prevalence increases with age. About half of the population over the age of 65 years and up to 90% of individuals over the age of 80 years are affected by presbycusis (Cruikshanks et al., 2010; Lin et al., 2011c). The consequences of hearing impairment can be far reaching, commonly affecting not only the hearing impaired person, but also their communication partners, primarily significant others (SOs), and social networks. According to the World Health Organization’s International Classification of Functioning, Disability, and Health (ICF) (WHO; World Health Organization, 2001) communication disability due to hearing impairment is an outcome of interactions between sensory impairment and participation in life. For instance, hearing impairment often makes it difficult to participate in social and cultural activities due to a restricted ability to interact and communicate with peers. This can lead to withdrawal from activities and participation potentially resulting in feelings of loneliness and social isolation (Pronk et al., 2011).

As auditory perception sets the basis for oral communication, the contribution of the auditory system often is narrowed down to the term “hearing.” However, the concept should be disentangled and extended into more specific mechanisms that drive the stream of oral communication, that is *hearing*, *listening*, *comprehending*, and eventually *communicating* (World Health Organization, 2001; Kiessling et al., 2003). In the communication pathway *hearing* represents an important, rather passive function denoting the perception of sound. It is usually at this stage of sensory processing that hearing impairment is described by means of audiometry. *Listening*, *comprehending*, and *communicating* on the other hand are considered more complex processes that require active engagement of the individual(s) as well as fast interactions between sensory and cognitive processing. For example, *listening* to someone can be referred to as hearing with intention and attention. As such listening often demands the expenditure of mental effort, because cognitive resources including attention and executive functions (EFs) have to be invested for goal pursuit. Besides, the information must be received and decoded in a unidirectional manner in order to be able to derive and understand meaning. This step is described as *comprehension* and takes place throughout conversations with others. Finally, *communication* involves the conversational interactions between two or more people, while transferring information, meaning and intent bi-directionally. Given the described steps in the communication pathway, successful oral communication depends not only on the ability of hearing, but also requires listening and comprehending from all participants involved. One could understand a communication situation as a dynamic system that must be carefully balanced. Difficulties in either one component, such as one communication partner being hearing impaired, would require sensitivity and flexibility by means of adaptation of the system. To maintain the flow of a conversation and to avoid interruptions, when communication problems occur, strategies for compensation and repair need be activated immediately. Such strategies could include that the speaker repeats or rephrases what was said using loud and clear voicing, or that the hearing impaired person tries to concentrate more and activates additional mental resources (e.g., filling the gaps through context) or relies more on other modalities (e.g., visual cues for lip-reading; Lind, 2009; Lind et al., 2010).

## Executive Functions and Attention Steer Oral Communication

**FIGURE 1:**
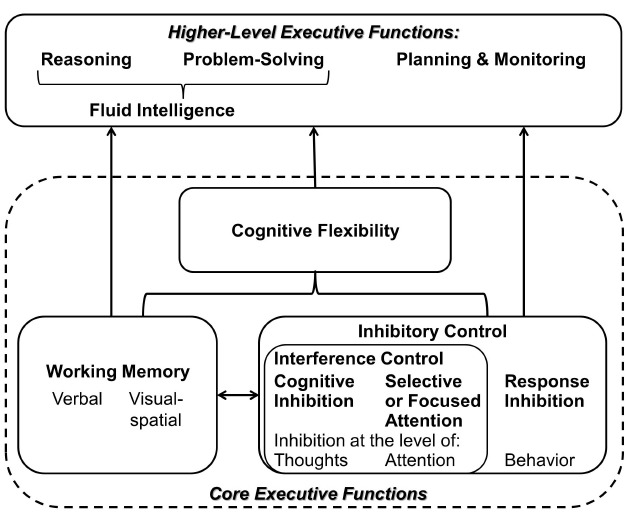
**Executive functions—a set of top-down mental processes (adapted from Diamond, 2013)**.

Oral communication requires concentration and paying attention, thus demanding specific top-down mental processes that are referred to as EFs (Miller and Cohen, 2001; Burgess and Simons, 2005; Diamond, 2013). These EFs enable the strategic handling of communicational intentions such as taking time to think before responding, considering unanticipated arguments, resisting the temptation to interrupt a communication partner, and staying focused throughout a conversation. Figure 1 shows EFs that have consistently been identified and that have been associated with a prefrontal-parietal neural network (Diamond, 2013). While there is inconsistency in the literature regarding the use of specific terms and the modeling of EFs, there is general agreement on three essential functions behind this network, namely *inhibitory and interference control*, *working memory*, and *cognitive flexibility* (Miyake et al., 2000; Miller and Cohen, 2001; Diamond, 2013). These core functions mediate higher order EFs such as reasoning, problem solving (the latter two being used synonymous with fluid intelligence), anticipation and planning. Overall, EFs describe the ability to guide attention, thought, and action in accord with goals or intentions as it is required in oral communication (Miller and Cohen, 2001).

The degree to which attention, EF and other cognitive resources have to be allocated and engaged for a specific listening goal is referred to as listening effort, which is especially reported under adverse listening conditions and for cognitively demanding listening tasks (Anderson Gosselin and Gagne, 2011; Picou et al., 2011; McGarrigle et al., 2014; and respective comments from Ronnberg et al., 2014; Wingfield, 2014). This is for instance the case, when auditory perception is compromised by distracting background noise, reverberant conditions, competing voices, and/or a degraded auditory signal due to a person’s hearing impairment (Arlinger et al., 2009). Under such circumstances, there is a high demand for core EFs, which is especially challenging in old age and will be outlined in more detail below (Erb and Obleser, 2013).

Firstly, *inhibitory and interference control* enable the selective allocation and reallocation of attention. Thus, it becomes for instance possible to focus on the voice of interest in a multi-talker environment, while suppressing other auditory streams. In hearing impairment, degraded signals trigger automatic, stimulus driven, bottom-up processing. Because they are more difficult to analyze they attract additional involuntary attention (Shinn-Cunningham, 2007; Shinn-Cunningham and Best, 2008). Consequently, it becomes more demanding to ignore or attend to specific stimuli driven by top-down goals and intentions (Posner and DiGirolamo, 1998). Also, it should be noted that older adults tend to develop difficulties in inhibition of distractions (Alain and Woods, 1999). While the ability of focusing attention usually remains intact in old age, there is strong evidence for an inhibitory-control deficit in aging (Gazzaley et al., 2005; Diamond, 2013). This age-specific difficulty is most probably taking its toll in complex communication situations and to an even greater extent in the presence of hearing impairment.

Secondly, the core EF of *working memory* (WM)—the ability to hold information in mind (maintain) and mentally work with it (manipulate) at the same time (Baddeley, 1992)—is key for speech understanding and communication (cf. new ELU-model; Ronnberg et al., 2013). WM allows one to relate things to each other over time, to consider alternatives, and to make decisions considering the past and the future. With regard to WM, evidence is in support of models that suggest a functional (maintenance vs. manipulation) as well as domain-specific organization (verbal vs. visual-spatial) in the frontal brain (Ullsperger and von Cramon, 2006). In the context of oral communication, verbal WM is necessary for comprehending speech, when meaning unfolds over the course of words and sentences. Nonetheless, visual-spatial WM can also play a role in the analysis of an auditory communication scene, as it facilitates the localization and segregation of speakers and other audio sources. Hearing impairment additionally loads on WM (e.g., when degraded information has to be put in context to derive its meaning; Ronnberg et al., 2013). Also here, a decline in WM capacity is common with age (e.g., Park, 1999) and constitutes an additional challenge for individuals with age-related hearing impairment. To a great extent this decline in WM seems to be due to the decline in inhibitory control (Hedden and Park, 2001). Moreover, a big overlap of age-related changes in speed of information processing and WM has been observed and controversially discussed (Salthouse, 1992; Zimprich and Kurtz, 2013). Inhibitory control and WM support each other. For example, in order to follow and participate in a conversation distracting thoughts and lines have to be disregarded and relevant information has to be retained.

Thirdly, it should be noted that the two previously discussed functions together provide the basis for a third core EF, which is *cognitive flexibility*. It describes the ability to change perspective regarding a problem, to be creative, to adjust to new demands, or to switch tasks according to priorities (Diamond, 2013). In general, cognitive flexibility also declines with age. For instance, in tasks that require switching between rules or response sets, older adults tend to slow down to maintain accuracy (Kray and Lindenberger, 2000; Cepeda et al., 2001). Older adults tend to recruit EFs in a rather reactive way in response to demands, whereas young adults tend to be anticipatory and proactively in recruiting EFs (Karayanidis et al., 2011).

## Cognitive Resources for Speech Comprehension

Central to oral communication is the ability to understand speech, which entails constant interactions between auditory and cognitive processing (Pichora-Fuller and Singh, 2006). Sounds continuously arrive at the ears via vibrations of air and are converted to linguistic representations in the brain (Craik, 2007). It is a bidirectional process taking in bottom-up information by using the perceptual system and conveying these inputs with top-down knowledge that has developed through experience (Pichora-Fuller, 2008a). Good quality of the signal facilitates speech understanding and better cognitive resources increase the chances to understand. In more detail, the bottom-up perspective is referred to as data-driven processing that involves mechanisms of conveying information from acoustic signals to phonemes, words, phrases and sentences. It is based on peripheral auditory processes that depend on the perceptual accuracy in coding and transferring acoustic information. Top-down effects, on the other side, are conceptually-driven cognitive processes that enable speech perception by linguistic context and expectation of the listener using the influence of memories and knowledge (Norman and Bobrow, 1975). Cognitive domains that apply for successful speech understanding primarily include speed of information processing (Review: Schneider et al., 2010), selective focused attention (e.g., Koelewijn et al., 2014), WM (e.g., Baddeley, 1992; Akeroyd, 2008) as well as semantic knowledge, namely language abilities and context integration (e.g., Pichora-Fuller and Singh, 2006; Zekveld et al., 2011).

In normal hearing individuals, the abilities to segregate, select, store, identify, and integrate information is often at risk in complex or adverse listening conditions. In case of hearing impairment and/or old age, additional challenges are introduced by compromised bottom-up information and/or decrements in top-down cognitive resources due to age-related changes (e.g., Bregman, 1990; Pichora-Fuller, 2003). Cognitive resources are generally limited and their processing “capacity” is assigned and flexibly shared between a number of tasks according to priorities (Moray, 1967; Kahneman, 1973; Wickens, 2008). In order to compensate for auditory deficits, hearing impaired listeners must invest more cognitive resources, for instance in order to follow a conversation. This is typically perceived as effortful by the listener. Also, these resources might otherwise be available for parallel tasks. In demanding listening situations, cognitive resources, such as rapidly switching attention and suppressing interfering sounds are additionally needed to extract the speech signal from competing sound sources and then to match it to mental representations of the phonological and semantic long term memory (cf. new ELU-model; Ronnberg et al., 2013). Consequently, less cognitive capacity is reserved and available for additional processes such as maintenance and manipulation of novel auditory information in WM or establishing episodic memory traces (Tun and Wingfield, 1999; Wingfield and Tun, 2001; Rudner et al., 2011; Mishra, 2014; Rudner and Lunner, 2014). In other words, speech understanding under adverse conditions takes up more cognitive capacity, firstly to decode the speech signal and secondly to comprehend it in order to be able to communicate, respectively. At this level, typical age-relevant cognitive declines in speed of information processing, inhibitory and interference control, WM capacity, and/or mental flexibility described earlier, may contribute even more to communication difficulties for the listener. Nevertheless, some of the above mentioned age-related challenges might be compensated if context information becomes available. For example, it has been shown that older people have a broader semantic knowledge and vocabulary, wider social experiences in a variety of communication situations, and make better use of prosody and context compared to younger individuals (Pichora-Fuller and Singh, 2006; Pichora-Fuller, 2008b).

Overall, speech understanding is realized through a widespread neural circuit that is mapped as a dynamic temporo-frontal network in the brain. Bottom-up information arrives at the auditory cortex within the temporal lobe and is directed to higher-order brain regions of the frontal cortex along multiple long-range language connections specified by ventral and dorsal pathways (Friederici, 2012). The ventral pathway is associated with the processing of sound-to-meaning and has been suggested to map acoustic speech signals onto lexical conceptual representations. The dorsal stream, on the other hand, is linked to the processing of sound-to-action and has been proposed the role of mapping signals onto articulatory motor representations (Hickok and Poeppel, 2007). More specifically, language-related brain areas typically comprise Broca’s area in the inferior frontal gyrus, Wernicke’s area in the superior temporal gyrus, and also parts of the middle temporal gyrus and the inferior parietal regions (Friederici, 2011). In this respect, the temporal cortex plays an important role for oral communication, given that this is the center where further connections for higher order processing are linked enabling the integration of attention, memory, and context for understanding speech. Interestingly enough, brain imaging studies have shown that with increasing age physiological changes in the healthy brain may become relevant for the integration of different cognitive resources in speech understanding under challenging listening conditions. These changes include reduced connectivity of neurons and thus interactions between brain regions; moderate loss of brain mass especially in the prefrontal cortex, medial temporal cortex (esp. hippocampus) or caudate nucleus; as well as changes in neurotransmitter systems such as the dopaminergic systems (e.g., Raz, 2005; Park and Reuter-Lorenz, 2009). Yet, literature has also shown that compensatory effects in old age as mentioned earlier are also reflected by brain activation patterns. A more extensive brain activity has been observed when listeners engaged in additional top-down context-driven processing (Davis et al., 2005). Primarily, activations in areas of the prefrontal and parietal cortices during listening in adverse conditions suggest increased functional connectivity between high-order cortical areas and indicate the allocation of additional, especially executive resources for semantic processing (Obleser et al., 2007). These widespread activations support compensatory processing in old age (Cabeza et al., 2002).

As there is a close association of hearing impairment and cognitive decline in old age, several explanations have been proposed and are debated (Li and Lindenberger, 2002; Lin et al., 2011a,d). Importantly, none of the explanatory models are exclusive, but instead could be coexistent. Namely, it has been hypothesized that sensory and cognitive decline in old age share their pathologic etiology and have a “common cause.” Also, the described interaction of hearing impairment and cognitive load in the sense of resource competition and limited capacity could explain this association. Last but not least, social and psychological factors have to be taken into account as the interaction of hearing and cognition could be mediated through those.

## Social Resources and Consequences

Considering that communication takes place between two or more individuals and in the context of culture and society, it is influenced by shared and unshared patterns of action, meaning, and values. These phenomena are intensively studied in social psychology with regard to intrapersonal (e.g., self-concept and social cognition) and interpersonal processes (e.g., social influence, group dynamics, attractions, and generation gap; e.g., Tesser and Schwarz, 2000; Fletcher and Clark, 2002). One’s thoughts, feelings, and behaviors are influenced by the presence of others and interaction with others. Therefore, difficulties in communication that are driven by hearing impairment may have significant consequences concerning the sense of security in everyday life, quality of life, social and emotional functioning as well as psychological wellbeing (e.g., Strawbridge et al., 2000; Nachtegaal et al., 2009, 2012; Danermark et al., 2010; Stam et al., 2013, 2014; Hogan et al., 2015). It is evident that poor hearing leads to communication impairments that may result in social isolation and may mediate disadvantageous health and functional consequences (Berkman et al., 2000; Uchino, 2006). Also, it is hypothesized that withdrawal from social participation may put hearing impaired people at risk for more rapid cognitive decline (Uhlmann et al., 1989; Gates et al., 2010, 2011; Lin et al., 2011a,b). In connection with this, the role of communication partners, especially SOs, in hearing impairment has gained interest during recent years. This is particularly evident in the WHO’s ICF classification (World Health Organization, 2001) of the effect of hearing impairment on SOs as a third-party disability. Third-party disability is described to occur when the SO does not have a hearing impairment themselves, but experiences activity limitations and participation restrictions as a result of their partner’s hearing impairment (Scarinci et al., 2009, 2012). SOs are reported to experience a restricted social life, increased burden of communication, and poorer quality of life and relationship satisfaction (Kamil and Lin, 2015). Treatment of hearing impairment that typically comprises hearing aids, cochlear implants, and audiological rehabilitation programs targeting the hearing impaired person, tend to also improve quality of life, communication, feelings toward the hearing impaired person, and activity participation of the SO (Kamil and Lin, 2015).

## Concluding Remarks

Modern audiology has extended its focus from hearing to considerations of cognitive processes, aging effects and social factors in order to address the communication problems of hearing impaired individuals and to meet their expectations. In recent years, great insight has been gained into this interdisciplinary field of study. For instance research has taken into account aspects of neuro-cognitive mechanisms, age-related decrements and compensatory strategies, as well as the role of SOs and the social network related to successful oral communication and rehabilitation. Nevertheless, there is still great potential for applying this knowledge as a matter of course in aural rehabilitation (e.g.,Ekberg et al., 2014, 2015; Hickson et al., 2014) and translating it into services and products to the benefit of the hearing impaired (e.g., Lunner et al., 2009; Pichora-Fuller et al., 2013).

### Conflict of Interest Statement

The authors declare that the research was conducted in the absence of any commercial or financial relationships that could be construed as a potential conflict of interest.
